# Change-point models for identifying behavioral transitions in wild animals

**DOI:** 10.1186/s40462-023-00430-0

**Published:** 2023-10-20

**Authors:** Kathleen P. Gundermann, D. R. Diefenbach, W. D. Walter, A. M. Corondi, J. E. Banfield, B. D. Wallingford, D. P. Stainbrook, C. S. Rosenberry, F. E. Buderman

**Affiliations:** 1 Department of Ecosystem Science and Management, Pennsylvania State University, University Park, PA USA; 2 U. S. Geological Survey, Pennsylvania Cooperative Fish and Wildlife Research Unit, Pennsylvania State University, University Park, PA USA; 3 Pennsylvania Cooperative Fish and Wildlife Research Unit, Pennsylvania State University, University Park, PA USA; 4 Pennsylvania Game Commission, Harrisburg, PA USA

**Keywords:** Change-point models, Deer, Elk, Hidden Markov model, Movement behavior, Parturition, Telemetry data

## Abstract

Animal behavior can be difficult, time-consuming, and costly to observe in the field directly. Innovative modeling methods, such as hidden Markov models (HMMs), allow researchers to infer unobserved animal behaviors from movement data, and implementations often assume that transitions between states occur multiple times. However, some behavioral shifts of interest, such as parturition, migration initiation, and juvenile dispersal, may only occur once during an observation period, and HMMs may not be the best approach to identify these changes. We present two change-point models for identifying single transitions in movement behavior: a location-based change-point model and a movement metric-based change-point model. We first conducted a simulation study to determine the ability of these models to detect a behavioral transition given different amounts of data and the degree of behavioral shifts. We then applied our models to two ungulate species in central Pennsylvania that were fitted with global positioning system collars and vaginal implant transmitters to test hypotheses related to parturition behavior. We fit these models in a Bayesian framework and directly compared the ability of each model to describe the parturition behavior across species. Our simulation study demonstrated that successful change point estimation using either model was possible given at least 12 h of post-change observations and 15 min fix interval. However, our models received mixed support among deer and elk in Pennsylvania due to behavioral variation between species and among individuals. Our results demonstrate that when the behavior follows the dynamics proposed by the two models, researchers can identify the timing of a behavioral change. Although we refer to detecting parturition events, our results can be applied to any behavior that results in a single change in time.

**Supplementary Information:**

The online version contains supplementary material available at 10.1186/s40462-023-00430-0.

## Introduction

Knowing the vital rates for wildlife populations of management or conservation concern is critical for determining the best management actions and assessing their outcomes. For example, vital rates can inform quotas and license sales [3, 40] and identify resources associated with survival and reproduction, which can guide habitat management [24, 56]. Birth and death rates impact population dynamics, such as recruitment, age structure, and population growth [14, 52]. The contribution of the birth rate to population dynamics is even greater for species with relatively constant adult survival [28]. One of the most direct ways to estimate birth rates and fecundity is to identify parturition events [17, 28, 52]. The most accurate methods for determining parturition rates include serum progesterone level tests [59], the use of ultrasonography [1], and vaginal implant transmitters (VITs) [6], however, these methods are expensive and invasive.

Parturition is accompanied not only by morphological [15], physiological [58], and hormonal changes [13] but also by behavioral shifts [60]. Females across taxa exhibit complex behavioral shifts during the last days of pregnancy and immediately following the parturition event [7, 48, 57]. These changes in behavior may manifest as changes in space use and movement-related quantities. For example, a change in space use may be identified as a species moving their geographic core use area prior to parturition, such as parturient brown hyenas (*Parahyaena brunnea*) isolating themselves from the group to give birth in natal dens [38, 49] and humpback whales (*Megaptera novaeangliae*) moving away from the pod during the birthing process [53]. Other species may alter their movement in terms of total area used or traversed and can be identified as quantifiable changes in movement metrics. For example, movement rates of female migratory caribou (*Rangifer tarandus*) are lower in females that gave birth compared to those that did not [26, 43].

Given the large number of wildlife studies using satellite telemetry devices to monitor individual movement [34, 42], these behavioral changes may provide researchers with an opportunity to indirectly detect parturition. The use of location monitoring technology such as global positioning system (GPS) technology, very high frequency (VHF) telemetry, and satellite tracking through the Argos system [41, 42, 61] can yield data that could help answer a wide breadth of questions regarding unobserved behavioral activity [4, 21], resource selection [5, 8], and other spatial and temporal patterns [2, 25]. As the accuracy and longevity of monitoring devices improves [61], researchers have been able to expand inference on wild species and systems to include detecting unobserved behaviors based on changes in fine-scale location data. Numerous studies have documented these parturition-related changes in movement by telemetering individuals and monitoring their locations [26, 38, 48, 62].

Previous work has demonstrated that fine-scale location data can identify behavioral shifts related to parturition in a single individual [62]. However, if parturition results in predictable changes in either space use or movement metrics across individuals in a population, the timing of parturition could be detected solely using location data obtained via telemetry collars for a majority of individuals. Utilizing location data directly could decrease the resources needed to monitor parturition rates and allow managers to identify pregnant individuals and the areas used by these individuals without relying on costly, invasive procedures or time-consuming field monitoring. Additionally, detection of parturition events using location data could assist researchers in determining real-time birthing locations for studies in which the neonate must be located immediately after birth [44].

Many studies in the last decade have developed various methods to identify parturient individuals and parturition events using movement metrics derived from location data [7, 20, 24, 44, 48, 62]. Other methods have been developed to identify when a behavioral change manifests in physical locations [31, 37], but few of these techniques have applied to identifying parturition timing or status [62]. Many of these methods include change-point models. Change-point models are ideal for identifying a known or fixed number of transitions in a sequence of time steps where a sudden and distinct change in the response variable occurs [31, 33, 62]. Hidden Markov models with an absorbing state could also be used to handle scenarios in which one-transition (such as parturition) occurs, however they can be difficult to implement using standard statistical software. Overall, few of these studies had validation data on the exact timing of the event of interest or pre-defined what would constitute a successful identification of an event. Without validation data, the success of models in determining when parturition occurred cannot be confirmed or quantified. Even when validation data are available, it is important to define a successful model outcome that is relevant to the objective (e.g., post hoc identifying parturient individuals vs locating a birthing site in real time). A pre-determined definition of success relevant to the ecological or logistical issue at hand allows researchers to objectively judge the performance of the proposed approach. Validation and model success are important to incorporate as movement metric and location-based change represent hypotheses about how individuals behave at parturition, and a direct comparison between the two behaviors has not been made.

To address these hypotheses, we developed two change-point models that capture different parturition-related changes in movement behavior: a location-based model and a movement metric-based model to identify the timing of these events. Comparing the two manifestations of behavioral shifts is important as a change in physical locations does not necessarily result in a change in movement metrics, and vice versa. For example, relocating a core area can be achieved without a detectable change in movement metrics, such as step lengths (the straight-line distance between two points) and turning angles (the change of direction between three successive steps). Meanwhile, the geographic location of the core-use area may not change, but if the core area becomes smaller post-parturition, then quantities such as step length and turning angle must necessarily change.

To test the ability of our change-point models to detect single behavioral shifts, such as parturition, we conducted a simulation study in which we varied the sampling effort (duration and frequency of observations). We used the simulation study to determine the optimal sampling efforts for detecting behavioral events that manifest as either a change in home-range location or derived movement metrics. We then applied our models to a case study of two ungulate species, white-tailed deer (*Odocoileus virginianus*) and Rocky Mountain elk (*Cervus canadensis nelsoni*), to test proposed hypotheses about parturition-related behavior in individual ungulates with known timing of parturition events. Species-specific behavior may result in different models being better at detecting parturition-related changes in movement [44] and there may be large amounts of individual variation in movement behavior [54]. Our research contributes not only to our understanding of among-species variation in parturition-related movement behavior but also provides guidance for researchers interested in determining the timing of single behavioral change events.

## Methods

### Modeling framework

The location-based and movement metric-based change-point models used the same structure, in which the observation at time *t* ($t = 1, \ldots , T$) arose from a mixture of two distributions, detailed further in Additional file 1: Appendix A. By using a spike and slab prior [36], the model could fail to detect a change, which would mean the change point (τ) was set to zero and the observations would only arise from one of the two distributions. When a change was detected, the change point ($\tau$) was modeled as a uniformly distributed categorical random variable with a span from 1 to *T*, where T is the total number of observations for an individual.

All models were fit to the simulation and case study data using program R [51] and the NIMBLE package [23]. We made inference on 200,000 Markov chain Monte Carlo (MCMC) iterations following a burn-in period of 100,000 iterations and thinned to every four iterations. We determined convergence through a visual check of posterior distributions and the Geweke diagnostic ( >|2| was not converged, [30]). For individuals that did not converge under these conditions, we refit the models using 500,000 MCMC iterations following a 250,000-iteration burn-in period and thinned every four iterations. We used a single chain due to the possibility of label-switching, which does not change the estimated parameters but complicates combining inference across chains [39]. If multiple chains are desired, one could prevent label switching by restricting mean parameters across the two states relative to each other through a set prior.

#### Location-based change-point model

The Location-based Change-Point Model (LCPM) is based on the 2-dimensional spatial location of the individual at time step *t* (${\mathbf{y}}_{t}$). To facilitate setting priors, we centered the easting and northing for each individual according to their respective means. This allowed us to use a single value, 0, as the mean location for all individuals spread across Pennsylvania. The locations were distributed as a mixture of two multivariate normal distributions

1 $$\begin{aligned} {\mathbf{y}}_{t} & \sim \left\{ {\begin{array}{*{20}l} {{\mathcal{N}}\left( {{{\varvec{\upeta}}}_{t1} ,{{\varvec{\Sigma}}}} \right)} \hfill & {{\text{if}}\;t < \tau } \hfill \\ {{\mathcal{N}}\left( {{{\varvec{\upeta}}}_{t2} ,{{\varvec{\Sigma}}}} \right)} \hfill & {{\text{if}}\;t \ge \tau } \hfill \\ \end{array} } \right. \\ \tau & \sim {\text{Categorical}}\left( {{\varvec{\uppsi}}} \right). \\ \end{aligned}$$

The multivariate normal distributions were parameterized by their time-varying expected location in space (${{\varvec{\upeta}}}_{ti} ,\;{\text{for}}\;i = 1, 2$), which varied depending on the relative value of the time step to the change point (pre- or post-change state) and a covariance matrix (${{\varvec{\Sigma}}}$). We modeled ${{\varvec{\Sigma}}} = \sigma^{2} {\mathbf{I}}$ where the prior for $\sigma^{{}}$ was modeled as Uniform(0, 10^7^), which did not vary depending on the state. To model the expected location at each time step, we used an autoregressive model of order one (AR(1)) where the location at time t, ${{\varvec{\upeta}}}_{t.}$, depended on the location at time t − 1, ${\varvec{y}}_{t - 1}$, but also included an attractor around a central location $\left( {{{\varvec{\upmu}}}_{i} ,\;{\text{for}}\;i = 1, 2} \right)$ to account for stationarity around a geographic centroid:

2 $$\begin{aligned} & {{\varvec{\upeta}}}_{t1} = {\mathbf{M}}{*} {\varvec{y}}_{t - 1}^{\prime } + \left( {{\mathbf{I}} - {\mathbf{M}}} \right){*}{{\varvec{\upmu}}}_{1}^{\prime } \\ & {{\varvec{\upeta}}}_{t2} = {\mathbf{M}}{*}{\varvec{y}}_{t - 1}^{\prime } + \left( {{\mathbf{I}} - {\mathbf{M}}} \right){*}{{\varvec{\upmu}}}_{2}^{\prime } . \\ \end{aligned}$$

**Fig. 1:**
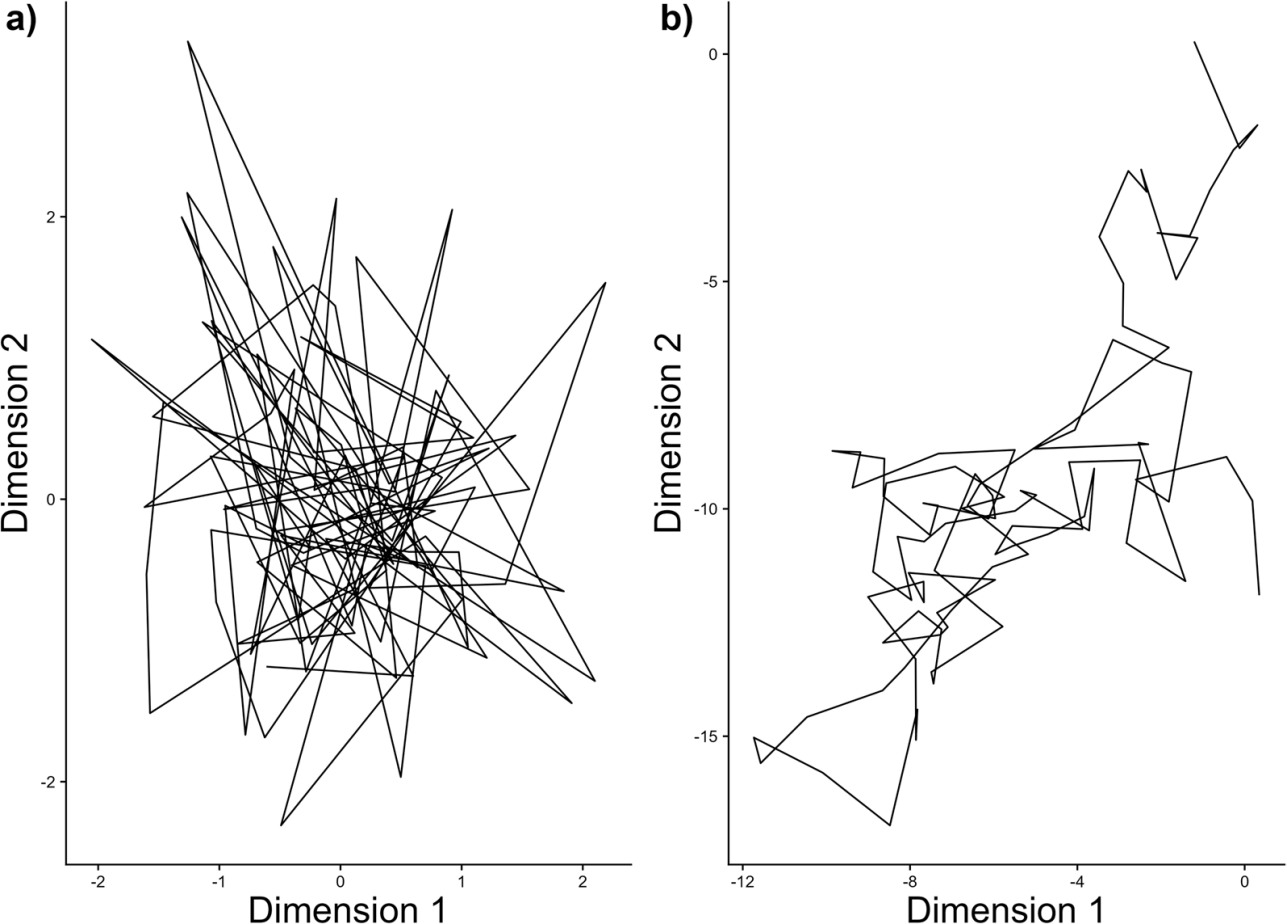
An illustration of two possible movement trajectories. We simulated a two-dimensional AR(1) process, described in Eq. (2), with the geographic centroids arising from a mean of c(0,0). In panel **a** we fixed the autocorrelation parameter, ρ, to 0. Under these circumstances, the locations are heavily influenced by the geographic centroid. In panel **b** we fixed ρ to 1, and the expected value of a location is based on the location at the previous time step with no attraction to the geographic centroid

Each geographic centroid, which is the component that varied between the two states, was modeled as ${{\varvec{\upmu}}}_{i} \sim{\mathcal{N}}\left( {0,{{\varvec{\Sigma}}}_{{{\varvec{\upmu}}}} {\mathbf{I}}} \right)$ for *i* = 1, 2, where ${{\varvec{\Sigma}}}_{\mu }$ = 5000*I. The degree of autocorrelation was estimated via the propagator matrix, ${\mathbf{M}} = \rho {\mathbf{I}}$, where $\rho$ is the autocorrelation parameter and has support of -1 to 1. While ${\mathbf{M}}$ acts directly on the previous location, it also indirectly affects the impact of the geographic centroid. As $\rho$ approaches one, the model simplifies to an "intrinsic" conditional autoregressive model (ICAR; [35]), because $\left( {{\mathbf{I}} - \rho {\mathbf{I}}} \right)$ goes toward zero and nullifies the attractor (Fig. 1a). In contrast, when $\rho$ approaches zero, the location at time *t* becomes less dependent on the location at time *t* − 1 and more dependent on the location of the attractor (Fig. 1b).

Preliminary results indicated that the autocorrelation parameter did not vary between states; therefore, we used a single autocorrelation parameter, ρ, arising from a Uniform(0, 0.8) prior, which allowed for positive autocorrelation while enforcing some dependence on a geographic centroid.

#### Movement metric-based change-point model

In the Movement Metric-based Change-Point Model (MMCPM), the observations of interest were turning angles and step lengths. Turning angles and step lengths are metrics widely used to characterize behavioral states in animal movement modeling [45, 47]. Turning angles capture tortuosity, and step lengths capture movement speed (given consistent time between observations). As above, we used $\tau$ to represent the change point across the models for turning angles and step lengths. We specified the turning angle at time *t* as

3 $$a_{t} \sim \left\{ {\begin{array}{*{20}l} {\begin{array}{*{20}l} {{\text{wrapped}}\;{\text{Cauchy}}\left( {\gamma_{1} ,\kappa_{1} } \right)} \hfill & {{\text{for}}\;t < \tau } \hfill \\ {{\text{wrapped}}\;{\text{Cauchy}}\left( {\gamma_{2} ,\kappa_{2} } \right)} \hfill & {{\text{for}}\;t \ge \tau } \hfill \\ \end{array} } \hfill \\ \end{array} .} \right.$$

The wrapped Cauchy distribution was parameterized using location ($\gamma_{i}$ for $i = 1, 2$), and scale ($\kappa_{i}$ for $i = 1, 2$), which varied depending on the current state (pre- or post-change) at time $t$. We used the wrapped Cauchy distribution to describe turning angles because it is a circular distribution, similar to a wrapped normal or von Mises distribution, but allows for the distribution to become uniform on a circle [35], as the scale parameter approaches zero, which would mean an individual is equally likely to move in any direction. To match the support for turning angles, which is in radians, the prior for the location parameter was modeled as Uniform($- {\uppi }, {\uppi }$), and the prior for the scale parameter was modeled as Uniform(0, 1).

The model for step length followed the same mixture-model structure; however, we found that different distributions were needed to describe the movements of deer and elk. We modeled elk step lengths as

4 $$\begin{array}{*{20}c} {\begin{array}{*{20}c} {{\text{s}}_{{{\text{elk}},t}} \sim \left\{ {\begin{array}{*{20}l} {\begin{array}{*{20}l} {{\text{Weibull}}\left( {\alpha_{1} ,\beta_{1} } \right)} \hfill & {{\text{for}}\;t < \tau } \hfill \\ {{\text{Weibull}}\left( {\alpha_{2} ,\beta_{2} } \right)} \hfill & {{\text{for}}\;t \ge \tau } \hfill \\ \end{array} } \hfill \\ \end{array} } \right.} \\ \end{array} } \\ \end{array} .$$

The Weibull distribution was parameterized using shape ($\alpha_{i} {\text{ for }}i = 1, 2$), and scale ($\beta_{i} {\text{ for }}i = 1, 2$) parameters, which varied depending on the current state at time $t$. The prior for the shape parameter was modeled as Gamma(0.001, 0.001), and the prior for the scale parameter was modeled as Uniform(0, 50). However, we found that the Weibull distribution did not adequately capture deer step lengths. The Weibull distribution tended to attribute the true parturition event to single observations of large step lengths that were interspersed throughout the observation period. These observations occurred more frequently in the deer dataset, which necessitated a less flexible model for step lengths. We instead used an exponential distribution such that

5 $$\begin{array}{*{20}c} {{\text{s}}_{{{\text{deer}},t}} \sim \left\{ {\begin{array}{*{20}l} {\begin{array}{*{20}l} {{\text{Exp}}\left( {\lambda_{1} } \right)} \hfill & {{\text{for}}\;t < \tau } \hfill \\ {{\text{Exp}}\left( {\lambda_{2} } \right)} \hfill & {{\text{for}}\;t \ge \tau } \hfill \\ \end{array} } \hfill \\ \end{array} } \right.} \\ \end{array}$$

where the exponential distribution was parameterized by rate ($\lambda_{i} {\text{ for }}i = 1, 2$), which varied depending on the current state at time $t$ and arose from a Gamma(0.001, 0.001).

### Simulation study

We first conducted a simulation study to determine if our proposed change-point models were able to detect behavioral shifts. We addressed three study design questions focused on a model's ability to detect the timing of the change correctly: the duration of observation following the event occurring, the fix interval, and the magnitude of change or consistency of change in geographic location or movement metrics, respectively (Additional file 2: Appendix B Tables S1, S2).

We were interested in these varying data scenarios given the range of objectives related to identifying a behavioral change. One objective may be to use incoming location data to decide when to initiate neonate search efforts because chances of capture decline with increased time after parturition [12]. Another objective may only be to determine parturition dates and locations post hoc. Each objective may require different monitoring strategies that vary in the frequency of locations and duration of monitoring. Therefore, if our models could detect behavioral shifts successfully, we wanted to provide guidance on estimating parturition solely from location data.

#### Simulating data

To simulate location data and movement metrics, we chose reasonable values given our observed data (Additional file 2: Appendix B Tables S3, S4). For example, we simulated data with $\rho$ fixed at 0.8, similar to preliminary estimates of the autocorrelation parameter among elk and deer in our case study.

To assess the effect of the duration of data obtained after the behavioral change, we simulated fifty complete datasets that contained locations 48 h prior to and after the change with a 15 min fix interval. We created additional truncated datasets from the complete dataset with post-change monitoring durations of 24, 12, 6, and 3 h (50 datasets each). To assess the effect of fix interval, we again simulated fifty complete datasets that contained locations 48 h prior and 24 h post-parturition with a 15 min fix interval. We thinned each complete dataset to both 30  and 60 min fix intervals.

A larger variation in observations and a smaller difference between the two states may make it more challenging to determine when the event of interest occurred. For the MMCPM, we determined four ways in which a difference in behavior might manifest: one with no change in step lengths or turning angles between pre-and post-parturition, one with a change in turning angles but not step lengths, one with a change in step lengths but not turning angles, and one with a change in both step lengths and turning angles. We simulated fifty datasets for each of these movement scenarios. For the LCPM$,{ }$ we used the largest difference between the estimated geographic centroids among individuals, as described above, and systematically decreased the difference to 75, 50, and 25% of the original distance (784.5, 523.0, and 261.5 m, respectively). We simulated fifty datasets for each of the  aforementioned differences with a fix interval of 15 min and 48 h of data pre-and post-parturition.

#### Model assessment

We first assessed if either LCPM or MMCPM detected a change point by calculating the mode of the posterior distribution of $\tau$. The spike and slab prior for τ allowed the model to be described by a single value if ${\uptau }$ was estimated to be the spike (set to zero). Therefore, a posterior mode of zero for ${\uptau }$ indicated that the models failed to detect a behavioral change. Given the mode was not zero, we then assessed the ability of each model to estimate the timing of parturition events for each simulated data set. For both models, we subtracted the true timing of the behavioral change from the posterior distribution of $\tau$. If the new posterior distribution was positive, our models overestimated the change point, and the true event occurred prior to the estimated change point. If this difference was negative, the true event occurred after the estimated change point. To quantify model accuracy, we a priori defined three levels of success for the models to accurately estimate the parturition event. A Level 1 success occurred when the upper and lower bounds of the 95% credible interval (hereafter CI) of the estimated change point both fell within ± 6 h of the true event. A Level 2 success occurred when the 50% CI fell within ± 6 h of the true event. A Level 3 success occurred when the posterior median fell within ± 6 h of the true event. If the upper and lower bounds of the 95% CI did not fall into one of the three levels of success, we considered this as an unsuccessful estimation of a change point. We chose 6 h as the window of interest to ensure the models could accurately capture the behavioral change at a fine scale. In the case study, we calculated the width of the 95% credible interval quantiles (hereafter quantile width) from the posterior distribution of the change point as a measure of estimate precision.

### Case study

#### Data collection and processing

The Pennsylvania Game Commission and the Pennsylvania State University captured 17 deer from January to April 2015–2017 and 37 elk from January to April 2020 in north-central Pennsylvania. Study area details can be found in Additional file 1: Appendix A. Adult females were fit with a vaginal implant transmitter (VIT; Vectronic Aerospace, Berlin, Germany) and a GPS satellite radio collar (GPS Plus, Vectronic Aerospace, Berlin, Germany). Deer and elk were handled according to protocols approved by The Pennsylvania State University Institutional Animal Care and Use Committee (Protocol No. 47054 and Protocol No. 01185). Further information on animal capture and data collection can be found in Additional file 1: Appendix A.

Our focus was on parturition events; therefore, we extracted individual location data from 3 days prior to and 4 days following the known parturition event (8 days total). We explored other pre- and post-parturition durations and found comparable results across durations [20, 48]. Because locations were not obtained at the same fix interval across study areas and years, we used the continuous-time functional movement model of Buderman et al. [11], fit using the ctmcmove R package [32], to interpolate locations to hourly intervals. All locations were in Universal Transverse Mercator (zone 17) easting and northing coordinates, and for the LCPM, we centered each individual's locations to their mean to more easily facilitate determining priors. We calculated step lengths and turning angles using the amt R package [55].

## Results

### Simulation study

#### Location-based change-point model

**Fig. 2:**
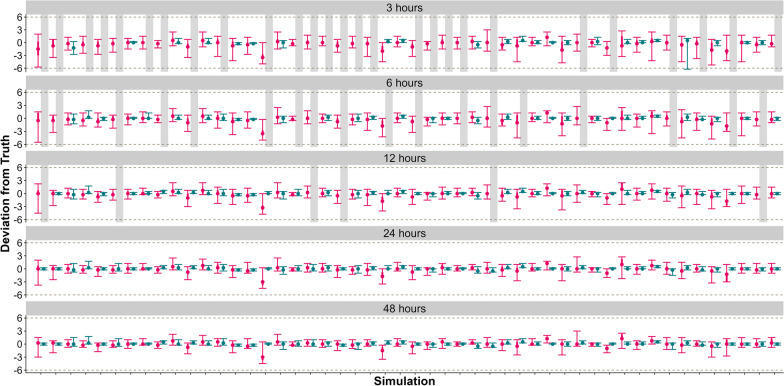
Fifty complete datasets of 48 h pre-and post-parturition at 15 min fix intervals were trimmed to 24, 12, 6, and 3 h of post-parturition data and fit to the Location-based Change-Point Model (LCPM; red) and Movement Metric-based Change-Point Model (MMCPM; blue). Gray, dashed lines represent ± 6 h of the true parturtition event. Gray rectangles represent simulations that did not detect a change, and red rectangles represent simulations that did not converge

The LCPM estimated a change point according to a Level 1 success for all datasets for each variation of post-parturition observation duration (Fig. 2, Table 1).

**Table 1:** Summary of our simulation study results (in percentages) for three design questions focusing on ability of the Location-based Change Point Model (LCPM) to correctly detect the timing of the change for and the magnitude of change in geographic locations (magnitude), duration of observation following the event occurring (duration), and fix interval (frequency)

Category	Magnitude				Duration					Frequency		
	100% Difference	75% Difference	50% Difference	25% Difference	48 H	24 h	12 h	06 h	03 h	15 min	30 min	60 min
Level 1	100	98	94	14	100	100	100	100	100	100	8	–
Level 2	–	2	–	24	–	–	–	–	–	–	92	–
Level 3	–	–	6	4	–	–	–	–	–	–	–	–
Not successful	–	–	–	56	–	–	–	–	–	–	–	–
No change detected	–	–	–	2	–	–	–	–	–	–	–	100
Did not converge	–	–	–	–	–	–	–	–	–	–	–	–

We simulated 50 datasets for each of these and summarized results based on a priori levels of success, whether no change point was detected, or lack of convergence

**Fig. 3:**
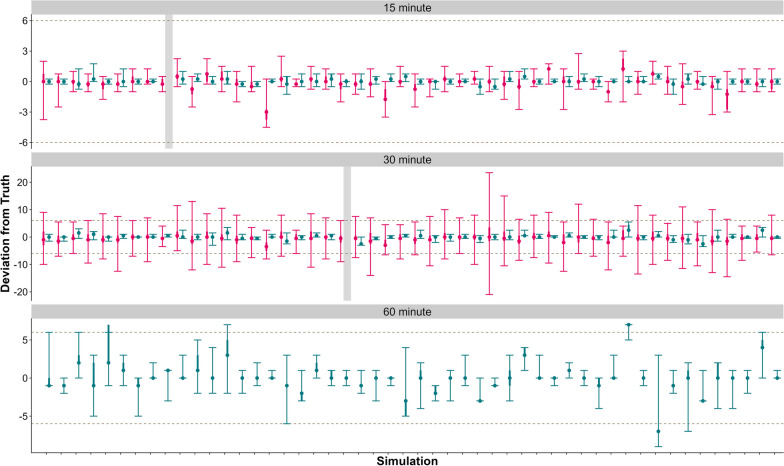
Fifty datasets of 48 h pre- parturition and 24 h post-parturition at a 15 min fix rate were thinned to 30 min and 60 min fix interval and fit each to the Location-based Change-Point Model (LCPM; red) and Movement Metric-based Change-Point Model (MMCPM; blue). Gray, dashed line represents ± 6 h of the true parturtition event. Gray rectangles represent simulations that did not detect a change. When thinned to a 60 min fix interval, LCPM did not detect a change point in any of the simulations. Note different scales for the y-axis

When we compared fix intervals, the LCPM estimated the change point according to Level 1 success for all the datasets when the fix interval was 15 min. However, when the datasets were thinned to 30 min and 60 min intervals, the LCPM did not estimate the change point as accurately (Fig. 3, Table 1).

**Fig. 4:**
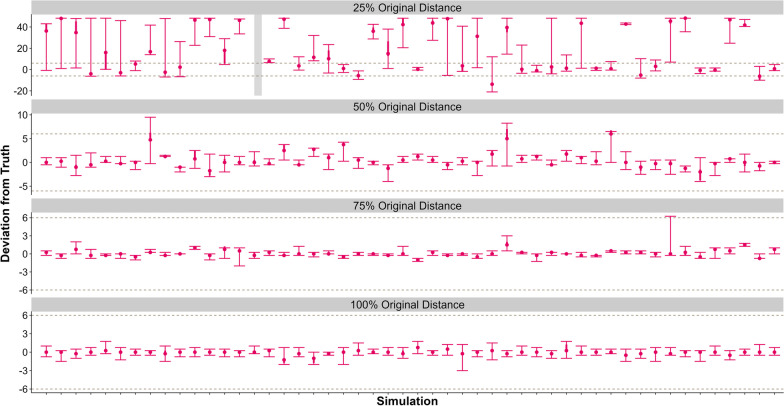
We simulated 50 datasets for varying distances between geographic centers (1026.0, 784.5, 523.0, and 261.5 m, respectively) and fit each to the Location-based Change-Point Model (LCPM; red). Gray, dashed lines represent ± 6 h of the true parturtition event. Gray rectangles represent simulations that did not detect a change, and red rectangles represent simulations that did not converge. Note different scales for the y-axis

Finally, for comparing the magnitude of change between pre- and post-parturition, the LCPM estimated the change point according to a Level 1 success for over 90% of the simulated datasets when the geographic centroids were 100%, 75%, and 50% of the maximum observed distance. Once the distance was reduced to 25% of the maximum observed distance, LCPM estimated the change point according to a Level 1, Level 2, and Level 3 14%, 24%, and 4% of the time respectively and did not successfully detect a change point in 56% of the datasets (Fig. 4, Table 1).

#### Movement metric-based change-point model

For differing post-parturition observation durations, the MMCPM estimated the change point according to Level 1 success for at least 90% of the datasets when there was at least 12 h of post-parturition data. However, once the observations were truncated to 6 and 3 h of data post-change, the ability of the MMCPM to estimate these changes decreased (Fig. 2, Table 2).

**Table 2:** Summary of our simulation study results (in percentages) for three design questions focusing on ability of Movement Metric-based Change-Point Model (MMCPM) to correctly detect the timing of the change for and the magnitude of consistency of change in movement metrics (step lengths and turning angles, SL and TA, respectively; magnitude), duration of observation following the event occurring (duration), and fix interval (frequency)

Category	Magnitude				Duration					Frequency		
	Difference in SL and TA	Difference in TA	Difference in SL	No difference	48 h	24 h	12 h	06 h	03 h	15 min	30 min	60 min
Level 1	100	98	–	–	100	100	88	64	44	98	98	92
Level 2	–	–	8	–	–	–	–	–	2	–	–	4
Level 3	–	–	–	–	–	–	–	–	–	–	–	–
Not successful	–	–	10	2	–	–	–	–	–	–	–	4
No change detected	–	2	82	96	–	–	12	36	54	2	2	–
Did not converge	–	–	–	2	–	–	–	–	–	–	–	–

We simulated 50 datasets for each of these and summarized results based on a priori levels of success, whether no change point was detected, or lack of convergence

When comparing the fix interval, the MMCPM estimated the change point according to a Level 1 success in 98% of datasets when the fix interval was 15 and 30 min. Once the fix interval was thinned to 60 min, the MMCPM slightly decreased in accuracy and estimated the change point according to Level 1 success in 90% of datasets (Fig. 3, Table 2).

**Fig. 5:**
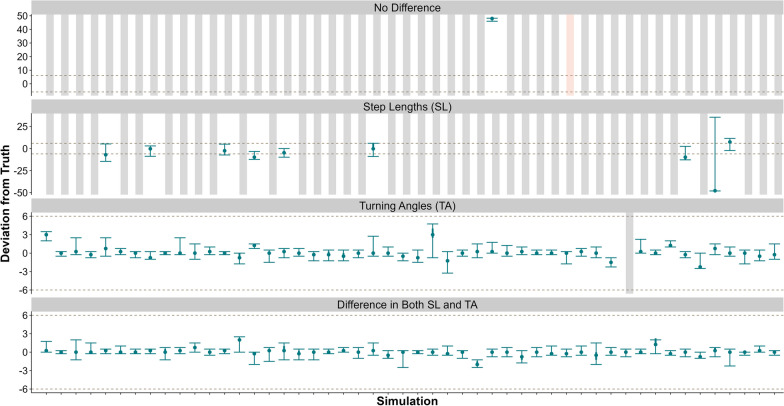
We simulated 50 datasets for four movement-metric situations, a difference in both turning angle and step lengths, a difference in just step lengths, a difference in just turning angles, and no difference in either before or after the change point and fit each to the Movement Metric-based Change-Point Model (MMCPM; blue). Gray, dashed lines represent ± 6 h of the true parturtition event. Gray rectangles represent simulations that did not detect a change, and red rectangles represent simulations that did not converge. Note different scales for the y-axis

Finally, when comparing the magnitude of change between pre- and post-event, the MMCPM performed best when a change in both step lengths and turning angles or just turning angles was present. In these scenarios the MMCPM estimated the change point according to Level 1 success for at least 98% of the datasets. When there was only a change in step lengths, the MMCPM did not detect a change occurred in 92% of datasets. When there was no change in either movement metric, the MMCPM did not detect a change in 92% of the datasets (Fig. 5, Table 2).

### Case study

#### Location-based change-point model

For deer, the LCPM did not consistently estimate when a change in geographic locations occurred. For eight of 17 individuals, the LCPM failed to detect a change in geographic locations. Of nine individuals for which the model detected a change, the LCPM estimated the change point according to a Level 2 success for one individual (average quantile width 29) and a Level 3 success for one (average quantile width 79; Table 3, Additional file 2: Appendix B Table S3 and Fig. S1).

**Table 3:** Summary of the number of individuals of white-tailed deer (*Odocoileus virginianus*) and Rocky Mountain elk (*Cervus canadensis nelsoni*; n = 17 and 37, respectively) for which the Location-based Change-Point Model (LCPM) detected a change or not

Species	Change point detected	Level of success	Number of individuals	Average 95% CI quantile width
Deer	Yes	Level 1	0	–
		Level 2	1	29
		Level 3	1	79
		Not successful	7	76.714
	No	–	8	–
	Did not converge	–	0	–
Elk	Yes	Level 1	0	–
		Level 2	0	–
		Level 3	0	–
		Not successful	37	16.875
	No	–	0	–
	Did not converge	–	0	–

If a change was detected, we calculated the 95% credible interval quantile width of the change point and averaged it within each species and level of success

**Fig. 6:**
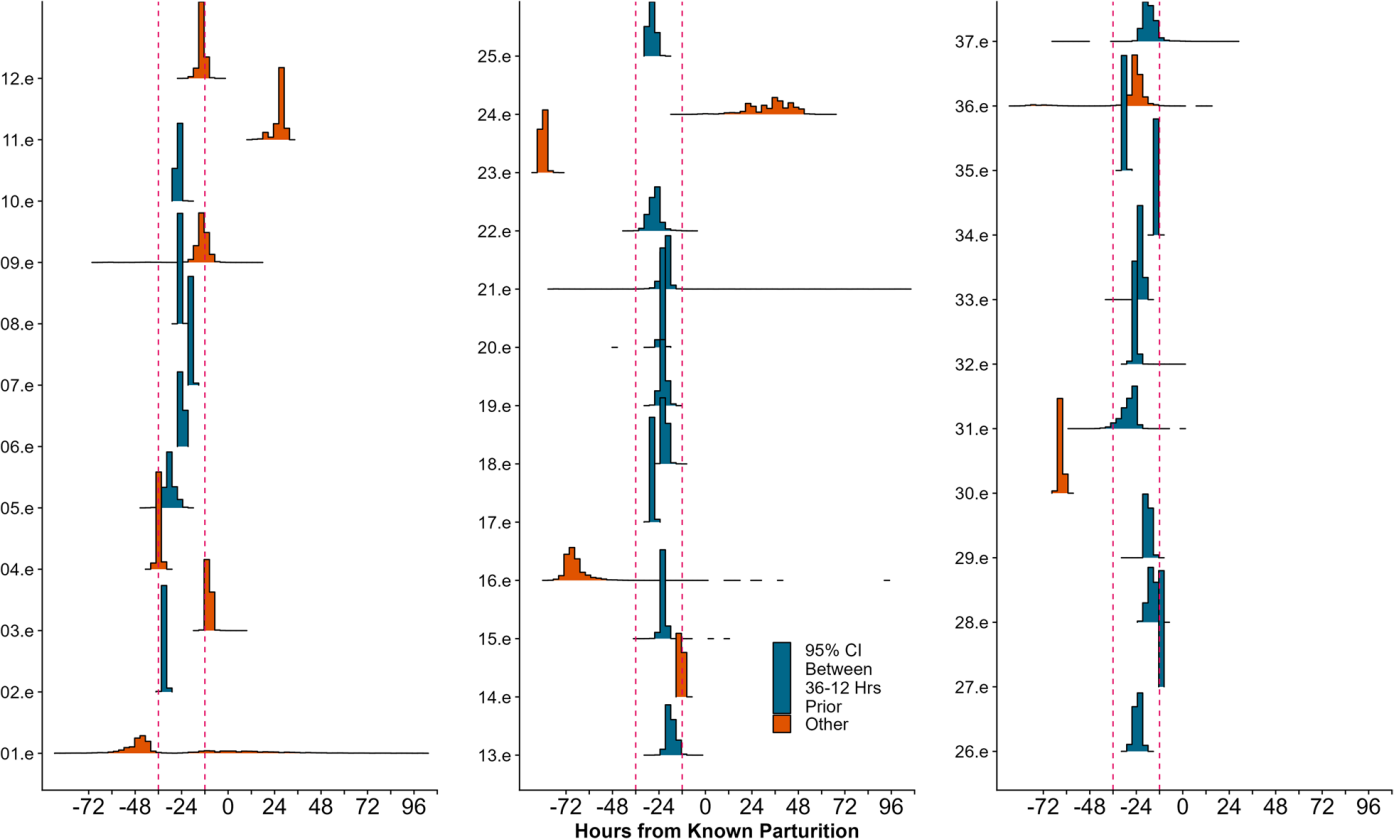
Results of the Location-based Change-Point Model (LCPM) fit to Rocky Mountain elk (*Cervus canadensis nelsoni*). For the 37 for which the LCPM detected a change we present the posterior distribution of the estimated change point centered on the known parturition event. The vertical, red dashed line represents 36 and 12 h prior to the known parturition event. Blue histograms represent individuals in which the 95% credible interval fell within this time frame

For elk, the LCPM detected a change in all 37 individuals (Table 3, Additional file 2: Appendix B Table S3, Fig. S2). Although no individuals met the criteria for our pre-defined levels of success, the model consistently estimated the change point between 36 and 12 h prior to the true parturition event (Additional file 2: Appendix B Fig. S2). In 25 individuals the 95% CI of the posterior distribution occurred within 36 to 12 h prior to true parturition (quantile width 4.64) and in four individuals, the 50% CI occurred within this range (quantile width 9.5; Fig. 6, Additional file 2: Appendix B, Fig. S3).

#### Movement metric-based change-point model

The MMCPM failed to converge for two deer, which were removed from the analysis. For 15 deer on which we made inference, the MMCPM did not detect a change point in nine individuals. In six individuals the MMCPM estimated that a change occurred, but none met the a priori categories of success (average quantile width 47.167; Table 4, Additional file 2: Appendix B Fig. S4).

**Table 4:** Summary of the number of individuals of white-tailed deer (*Odocoileus virginianus*) and Rocky Mountain elk (*Cervus canadensis nelsoni*; n = 17 and 37, respectively) for which the Movement metric-based Change-Point Model (MMCPM) detected a change or not

Species	Change point detected	Level of success	Number of individuals	Average 95% CI quantile width
Deer	Yes	Level 1	0	–
		Level 2	0	–
		Level 3	0	–
		Not successful	6	47.167
	No	–	9	–
	Did not converge	–	02	–
Elk	Yes	Level 1	6	5.2
		Level 2	3	11
		Level 3	1	18
		Not successful	27	8.615
	No	–	0	–
	Did not converge	–	0	–

If a change was detected, we categorized the ability of the model into three a priori levels of success based on the posterior distribution of the difference of the estimated change point and known parturition. Additionally, we calculated the 95% credible interval (CI) quantile width of the change point and averaged it across each species and level of success

We also observed large credible intervals, many spanning the support of the change point, and multi-modal posterior distributions (Additional file 2: Appendix B, Table S4, Fig. S4).

**Fig. 7:**
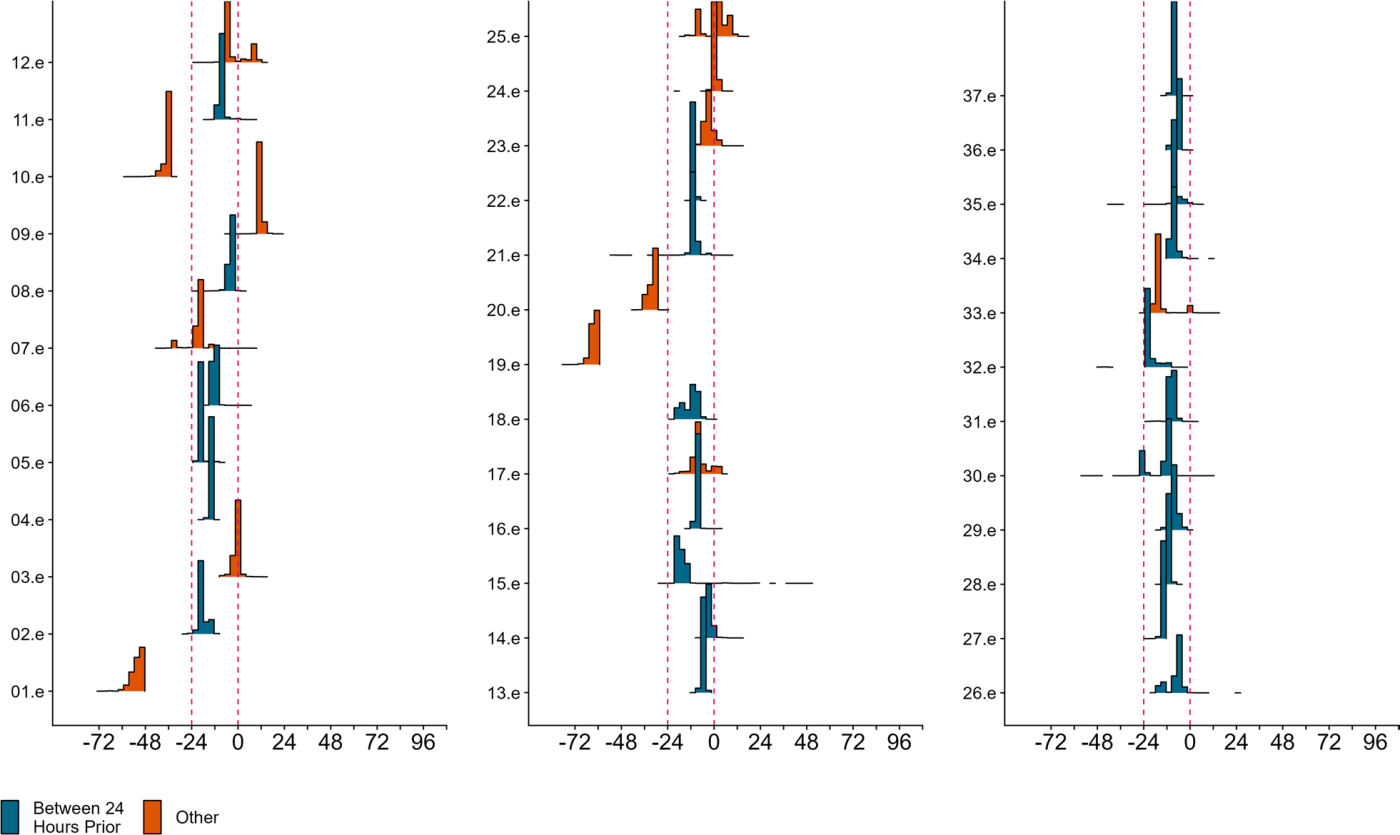
Results of the Movement Metric-based Change-Point Model (MMCPM) fit to Rocky Mountain elk (Cervus canadensis nelsoni). For the 37 for which the MMCPM detected a change, we present the posterior distribution of the estimated change point centered on the known parturition event. The vertical, red dashed lines represents 24 h prior to the known parturition event. Blue histograms represent individuals in which the 95% credible interval fell within this time frame

The MMCPM detected a change point in all 37 elk. Although 27 individuals did not meet one of the three a priori levels of success, many observed credible intervals were small (average quantile width 8.615) and in 24 individuals the 95% credible intervals of the estimated change point fell within the 24 h prior to the known parturition event (average quantile width 4.83; Fig. 7, Additional file 2: Appendix B, Table S4, Fig. S5). Six elk were classified as a Level 1 success, (average quantile width 5.2), three individuals were classified as a Level 2 success (average quantile width 11) and the remaining individuals was classified as a Level 3 success (average quantile width 18; Table 4, Additional file 2: Appendix B Table S4, Fig. S5). We observed multi-modal posterior distributions in some elk. However, the spans of the posterior distributions were small and six individuals were classified as a success (Additional file 2: Appendix B Figs. S5, S6.)

## Discussion

We developed a general Bayesian framework for fitting change-point models that can describe two likely manifestations of movement-related changes in behavior: changes in physical locations [49, 53] and movement metrics [26, 43]. Results from our simulation study indicated that changes related to parturition in ungulates could be detected in the absence of auxiliary validation data (e.g., VITs) given that individuals actually exhibit a change in location or movement behavior, therefore, depending on parturition detection objectives and individual behavior, our models can be used to reduce costs associated with current partition-detection methods.

Through our simulation study, we demonstrated that successful detection of a change point was possible, and we determined optimal data collection for when an individual exhibits the behavioral change described in the models. Due to constraints imposed by the battery power of telemetry devices, researchers must make a tradeoff between observation frequency (fix interval) and the duration of observation. The balance between fix interval and the duration of observation is essential when considering movement behavior because quantities, such as mean, maximum, and total distance moved, will vary as a function of fix interval [50]. Although temporally fine-scale data may identify behavioral changes soon after they occur, the increased frequency will lead to decreased battery life [46].

Given these potential sources of variation in the detection of a change point, our simulation study evaluated the ability of our two models to detect a given change under varying post-event sampling durations, fix intervals, and magnitude of behavioral changes. We determined successful detection of a change point was possible given a 15 min fix interval, at least 3 h of observation following a change for LCPM and at least 12 h for MMCPM. Moreover, for the MMCPM, successful detection of a behavioral change can occur with a 60 min fix rate and at least 24 h of observation following a change. The success of our simulation study demonstrates that when behavior follows the dynamics proposed by the two models, researchers can detect the timing of true behavioral change with current technology.

When we applied our change-point models to two ungulate species, not all individuals within a species exhibited movement behaviors captured by the models. This could be due to large behavioral variation across species and individuals. For deer, the LCPM failed to detect a change in a majority of the individuals and the ability to estimate a change was not consistent among individuals. These results indicate deer are not consistently changing their locations prior to or during parturition. In over 50% of the elk, however, the LCPM consistently identified and estimated a change within 12–36 h of parturition (Fig. 6, Additional file 2: Appendix B Fig. S3). Previous research has shown that elk may alter the locations they use prior to parturition as a potential predator avoidance strategy [29], and our models support this behavior by consistently estimating the parturition event 36–12 h prior to the true event.

Much like the LCPM, the MMCPM could not consistently or accurately estimate parturition events in deer. The failure of the MMCPM to capture parturition behavior of deer could be due to brief behavioral changes that are not able to be detected by the MMCPM. For example, females have been observed moving their fawns to a second location within 3 to 24 h after parturition and will only re-visit these secondary locations briefly at dawn and dusk to nurse [22]. While elk may exhibit a more detectable change in movement behaviors in the 24 h leading up to the birthing event. Thus, movement metrics, like step lengths and turning angles, may be more readily differentiated between pre-and post-parturition movements in elk than deer. Lack of success across deer in our case study indicates that, despite prior support in the literature, deer do not consistently follow the dynamics described by the two models, while elk do [18, 60]. Therefore, validation data on the event of interest should be used to determine if these models are a viable tool to identify the timing of an event of interest [10].

Based on change point analysis of telemetry data from the mother, locating neonates in real-time is unlikely to be successful given individual variation [54] and the potential absence of detectable behavioral changes. Additionally, our simulation study indicated the MMCPM would not be ideal for identifying parturition events in real time given that this model needs at least 12 h of post-parturition data to identify the change and additional time to mobilize and find the neonate. In combination with our results from the case study, the change in movement metrics was exhibited in the 24 h preceding the parturition event. The time needed for the MMCPM to detect behavioral changes would lead to decreased success of locating the neonate as time from parturition increases [12]. The LCPM, however, needs only 3 h of post-parturition data to identify behavioral change. If the change occurs prior to parturition, managers and researchers could be alerted ahead of time to a possible parturition event. After the behavioral change is detected, personnel could identify clusters of data points that might indicate a parturition event has occurred in real-time. For example, in our case study of elk, LCPM consistently underestimated the timing of parturition, which means that female elk initiated a move to the calving site 12–36 h before parturition (Fig. 6, Additional file 2: Appendix B Figs. S3, S4). The identified change point could be an indicator to initiate monitoring of the spatial distribution of the locations and begin preparing for a neonate search. Some collar manufacturers currently offer features such as virtual fencing, where researchers are notified when a collared individual exits or enters a preset polygon. Used in complement with LCPM this feature could aid in monitoring a collared individual following the change point. If managers and researchers are not interested in locating the neonate, but rather identifying the timing and location of parturition events (and the associated resources), MMCPM or LCPM could be used. The MMCPM can identify behavioral changes with less frequent data than the LCPM, which could extend the transmitter's battery life [9].

It is important to be cautious when assigning behaviors or events of interest from the change point estimated by a model [10]. The change-point framework simply detects when the most probable generating distribution transitions from one state to another. Researchers must use their ecological knowledge and expertise to apply biological significance to the estimated change. Wild animals are navigating a complex environment, and many unobserved variables, such as human activity [16], interactions with predators [27], or injury [19], can result in changes to movement behavior. For example, we detected several individuals for which the posterior distribution of the change point was bimodal (Additional file 2: Appendix B Fig. S7). Ecologically, this may indicate that multiple behavioral changes, potentially including parturition, occurred within the timeframe of interest. Incorrectly assigning a transition between statistical distributions to an event could lead to incorrect inference about the ecological process of interest [10]. Validation data can reduce erroneous behavioral assignments, but it is time-consuming and requires extensive resources.

However, when a behavioral change event is known to occur, our two change-point models successfully identified it under different monitoring and ecological scenarios. Therefore, these models could be used to identify the timing of parturition events, but only if the methods have been validated a priori. These methods and guidance can be applied in the future to other systems where single behavioral change occurs, such as migration, natal dispersal, or survival of offspring. Our change-point models provide a valuable tool for wildlife managers and researchers to monitor vital rates for populations of management and conservation interest.

### Supplementary Information

**Additional file 1.** Supplementary information on modeling framework, study area, and data collection and processing.

**Additional file 2.** Supplementary figures and tables for simulation and case study.

## Data Availability

Data and scripts are available on https://github.com/kpgund/change-point-datashare. All locations of deer and elk are centered on (0,0) to protect sensitive information.
