# Supplementary material for: Change-point models for identifying behavioral transitions in wild animals
Source: Mov Ecol. 2023 Oct 20;11:65. doi: 10.1186/s40462-023-00430-0 (PMC10589947; doi:10.1186/s40462-023-00430-0)
Supplement: Supplementary file 1 — Additional file 1. Supplementary information on modeling framework, study area, and data collection and processing. [file 40462_2023_430_MOESM1_ESM.docx]

## Supplementary information on modeling framework, study area, and data collection and processing

## APPENDIX A

Modelling framework

Both models used the same basic change-point structure, in which the observation at time *t* ($t = 1,\ldots, T$) arose from a mixture of two distributions. Unlike a state-space or hidden Markov model, where individuals can transition between behaviors (Zucchini et al., 2017), our change-point models allow for only one transition during the observation period; in hidden Markov model terminology the latent states would be considered pre-parturient and post-parturient. We allowed for only a single state transition because parturition in our study species can only occur once during the observation period. By using a spike and slab prior (Cappello et al., 2021), the model could also fail to detect a change, which would mean the observations could only arise from one of the two distributions. When a change was detected, the change point ($\tau$) was modeled as a uniformly distributed categorical random variable with a span from 1 to *T*, where T is the total number of observations. A general one-dimensional change-point model can be written as

$\begin{matrix} y_{t}\sim\left\{ \begin{matrix} \begin{matrix} \mathcal{N}\left( \mu_{1},\sigma_{1}^{2} \right) & \mathrm{for}t<\tau\\ \mathcal{N}\left( \mu_{2},\sigma_{2}^{2} \right) & \mathrm{for}t\geq\tau\end{matrix} \end{matrix} \right. \\ \tau\sim Categorical\left( \boldsymbol{\psi} \right) \end{matrix} \left( 1 \right)$,

where an observation at time *t* ($y_{t}$) arises from one of two normal distributions with different means ($\mu_{i}$ for $i = 1, 2$) and variances ($\sigma_{i}^{2}$ for $i = 1, 2$), depending on the position of the current time step relative to the change point. Below, we describe the location-based and movement metric-change-point models.

Study area

We monitored two species of ungulates, white-tailed deer and Rocky Mountain elk, throughout northcentral Pennsylvania. The area is characterized by cold, dry winters (mean temperature -1.44 degrees C) and hot, wet summers (mean temperature 21.56° C) (NRCC Summary Tables 2023). White-tailed deer were monitored in four study areas. The first two areas were in Rothrock (RR) and Bald Eagle (BE) State Forests in central Pennsylvania, United States, located in Centre, Mifflin, and Huntington counties. These state forests are part of the Ridge and Valley physiographic ecoregion, which is identified by the sandstone-capped ridges that run parallel to one another in a northeast-southwest direction, and the shale- and limestone-based valleys interwoven between the ridges (Nowacki and Abrams 1992). The elevation difference between valleys and ridges ranged between 400 and 700 meters (Cuff 1989). These two study areas are predominately forested, with the most dominant tree species being red and white oak (*Quercus* spp.), red maple (*Acer rubrum*), black birch (*Betula lenta*), black gum (*Nyssa sylvatica*), and hickory (*Carya* spp.). The understory layer is dense and contains many shrub species (*Vaccinium* spp., *Gaylussacia* spp., and *Kalmia latifolia*) (Van Buskirk 2020).

The remaining two study areas for deer were in Susquehannock State Forest in Potter County: Susquehannock North (SN) and Susquehannock South (SS). These two areas are north of the RR and BE State Forests and part of the Appalachian Plateau physiographic region (Cuff 1989). This area is characterized by plateaus interspersed with steep drainages and valleys. The elevation difference between the valleys and plateaus ranged between 220 – 800 m (Cuff 1989, Banfield and Rosenberry 2021). Much like RR and BE, SN and SS are predominately forested with dominant tree species of red maple, sugar maple (*Acer saccharum*), black cherry (*Prunus serotina*), and American beech (*Fagus grandifolia*). The elk were monitored in the Elk Management Area (Banfield and Rosenberry 2021), located in all of Cameron County and portions of Elk, Clinton, Potter, Clearfield, Tioga, Jefferson, Lycoming, and McKean counties. This area is also a part of the Appalachian Plateau and is similar to the white-tailed deer study areas in SN and SS State Forests.

Data collection and processing

The Pennsylvania Game Commission and the Pennsylvania State University captured deer from January to April 2015-2017 with rocket nets and Clover traps (Hawkins et al., 1968). We fit all adult (one years or older) female deer with a vaginal implant transmitter (VIT; Vectronic Aerospace, Berlin, Germany) and a GPS satellite radio-collar (GPS. Plus, Vectronic Aerospace, Berlin, Germany). The GPS collars and VITs were linked via ultra-high-frequency (UHF) radio signal. The GPS collar transmitted a satellite message once the VIT recorded a change in temperature and light. Once the message was received, we waited at least six hours to allow time for doe-fawn social bonding (Kilgo et al., 2012) before searching for the fawn. We confirmed that a parturition event had occurred by locating the coordinates sent by the GPS-transmitted message and conducted a visual search of the surrounding area to locate the neonate. If we could not find the neonate during the initial search, we conducted another search of the area at least six hours later. We programmed the GPS collars to obtain a location every three hours before the VIT was expelled and every 20 min for six weeks following the birthing event. We captured and confirmed pregnancy and parturition for 17 deer whose fawn survived for 48 hours. The time of the GPS-transmitted message was not recorded, and true parturition time was considered 8 hours prior to fawn capture.

We captured female elk from January to April 2020 through a combination of free darting and Clover traps (Thompson et al., 1989). We determined the pregnancy status of adult female elk through a rectal ultrasound examination and fit parturient individuals with VITs and GPS collars (Vertex Plus-3 Collar, Vectronic Aerospace, Berlin, Germany). Similar to deer, the GPS collar and VITs were linked via UHF radio signal and the GPS collar would transmit a message once the VIT recorded a change in temperature and lack of motion. Once the message was received, we waited at least three hours before starting a search for the calf and confirmed parturition by locating the neonate. We programmed the GPS collars to obtain a location every 13 h from the time of capture to 15 May 2020, after which the collars obtained a location every six h. The collars maintained this fix interval until the date the VIT was expelled, and the calf located. Once the calf was located, cow elk collars were reprogramed to obtain a location once every hour for six weeks. We captured and confirmed pregnancy and parturition for 37 elk whose calf survived for 48 hours. The time of the GPS-transmitted message was recorded for all elk, and true parturition was considered one hour after the message was received. We focused on three days prior to and four days following the known parturition event (eight days total), but compared different durations such as 5 days prior and 3 days post, 4 days prior and 2 days post, 3 days prior and 2 days post, 2 days prior and post and found similar results.

LITERATURE CITED

Banfield, J., and C. Rosenberry. 2021. Pennsylvania elk management plan (2020-2025).

Cappello, L., O. H. M. Padilla, and J. A. Palacios. 2021. Scalable Bayesian change point detection with spike and slab priors. arXiv. <http://arxiv.org/abs/2106.10383>. Accessed 18 Nov 2022.

Cuff, D. J. 1989. The atlas of Pennsylvania. Temple University Press.

Hawkins, R., L. Martoglio, and G. Montgomery. 1968. Cannon-netting deer. The Journal of Wildlife Management 191–195.

Kilgo, J. C., H. S. Ray, M. Vukovich, M. J. Goode, and C. Ruth. 2012. Predation by coyotes on white-tailed deer neonates in South Carolina. The Journal of Wildlife Management 76:1420–1430.

Nowacki, G. J., and M. D. Abrams. 1992. Community, edaphic, and historical analysis of mixed oak forests of the Ridge and Valley Province in central Pennsylvania. Canadian Journal of Forest Research 22:790–800.

*NRCC summary tables*. 2023. Northeast Regional Climate Center. Retrieved April 6, 2022, <https://www.nrcc.cornell.edu/regional/tables/tables.html/>.

Thompson, M. J., R. E. Henderson, T. O. Lemke, and B. A. Sterling. 1989. Evaluation of a collapsible clover trap for elk. Wildlife Society Bulletin 17:287–290.

Van Buskirk, A. 2020. Estimating the effects of changes in harvest management on white-tailed deer (*Odocoileus virginianus*) populations. Pennsylvania State University, MS Thesis.

Zucchini, W., I. L. MacDonald, and R. Langrock. 2017. Hidden Markov models for time series: an introduction using R, second edition. CRC Press.
