# Supplementary material for: Change-point models for identifying behavioral transitions in wild animals
Source: Mov Ecol. 2023 Oct 20;11:65. doi: 10.1186/s40462-023-00430-0 (PMC10589947; doi:10.1186/s40462-023-00430-0)
Supplement: Supplementary file 2 — Additional file 2. Supplementary figures and tables for simulation and case study. [file 40462_2023_430_MOESM2_ESM.docx]

## Supplementary figures and tables for simulation and case study

APPENDIX B

TABLES

Table 1: Parameter values used to simulate locations for datasets with varying durations of observations after parturition (durations), frequency of observations (frequency), and the distance between geographic centroids (magnitude) for the LCPM. The bold values indicate the set of values used for a comparison, while holding the other values constant.

| Location-based Change-Point Model | ρ | μ_1_ | μ_2_ | Distance (m) | Duration | Frequency |
| --- | --- | --- | --- | --- | --- | --- |
| Duration | 0.8 | [403.600, 383.725] | [-337.576, -354.091] | 1046.0 | **3 h** | 15min |
|  | 0.8 | [403.600, 383.725] | [-337.576, -354.091] | 1046.0 | **6 h** | 15min |
|  | 0.8 | [403.600, 383.725] | [-337.576, -354.091] | 1046.0 | **12 h** | 15min |
|  | 0.8 | [403.600, 383.725] | [-337.576, -354.091] | 1046.0 | **24 h** | 15min |
|  | 0.8 | [403.600, 383.725] | [-337.576, -354.091] | 1046.0 | **48 h** | 15min |
| Frequency | 0.8 | [403.600, 383.725] | [-337.576, -354.091] | 1046.0 | 24 h | **15min** |
|  | 0.8 | [403.600, 383.725] | [-337.576, -354.091] | 1046.0 | 24 h | **30min** |
|  | 0.8 | [403.600, 383.725] | [-337.576, -354.091] | 1046.0 | 24 h | **60min** |
| Magnitude | 0.8 | [403.600, 383.725] | **[-337.576, -354.091]** | **1046.0** | 48 h | 15min |
|  | 0.8 | [403.600, 383.725] | **[-152.282, -169.637]** | **784.5** | 48 h | 15min |
|  | 0.8 | [403.600, 383.725] | **[33.012, 14.817]** | **523.0** | 48 h | 15min |
|  | 0.8 | [403.600, 383.725] | **[218.306, 199.271]** | **261.5** | 48 h | 15min |

Table 2: Parameter values used to simulate step lengths and turning angles for datasets with varying durations of observations after parturition (durations), frequency of observations (frequency), and the magnitude of change (magnitude) for the MMCPM. The bold values indicate the set of values used for the comparison within a variable, while holding the other values constant.

|  | Turning Angle | | | | Step Length | | | |  | |
| --- | --- | --- | --- | --- | --- | --- | --- | --- | --- | --- |
| Movement Metric-based Change Point | γ_1_ | γ_2_ | κ_1_ | κ_2_ | α_1_ | α_2_ | β_1_ | β_2_ | Duration | Frequency |
| Duration | -0.037 | -2.367 | 0.72 | 0.19 | 1.561 | 1.168 | 0.159 | 0.064 | **3hr** | 15min |
|  | -0.037 | -2.367 | 0.72 | 0.19 | 1.561 | 1.168 | 0.159 | 0.064 | **6hr** | 15min |
|  | -0.037 | -2.367 | 0.72 | 0.19 | 1.561 | 1.168 | 0.159 | 0.064 | **12hr** | 15min |
|  | -0.037 | -2.367 | 0.72 | 0.19 | 1.561 | 1.168 | 0.159 | 0.064 | **24hr** | 15min |
|  | -0.037 | -2.367 | 0.72 | 0.19 | 1.561 | 1.168 | 0.159 | 0.064 | **48hr** | 15min |
| Frequency | -0.037 | -2.367 | 0.72 | 0.19 | 1.561 | 1.168 | 0.159 | 0.064 | 24hr | **15min** |
|  | -0.037 | -2.367 | 0.72 | 0.19 | 1.561 | 1.168 | 0.159 | 0.064 | 24hr | **30min** |
|  | -0.037 | -2.367 | 0.72 | 0.19 | 1.561 | 1.168 | 0.159 | 0.064 | 24hr | **60min** |
| Magnitude | **-0.037** | **-2.367** | **0.72** | **0.19** | **1.561** | **1.168** | **0.159** | **0.064** | 48hr | 15min |
|  | **-0.037** | **-2.367** | **0.72** | **0.19** | 1.561 | 1.561 | 0.159 | 0.159 | 48hr | 15min |
|  | -0.037 | -0.037 | 0.72 | 0.72 | **1.561** | **1.168** | **0.159** | **0.064** | 48hr | 15min |
|  | -0.037 | -0.037 | 0.72 | 0.72 | 1.561 | 1.561 | 0.159 | 0.159 | 48hr | 15min |

Table 3: Summary of LCPM in deer and elk. If a change was detected, we categorized the ability of the model into three *a priori* levels of success based on the posterior distribution of the estimated change point centered on the known parturition event. For each individual we present the 95% and 50% credible intervals (CI) for the posterior distribution of the estimated change point centered on the true parturition event and the quantile width for the 95% CI.

|  | | | | | | |
| --- | --- | --- | --- | --- | --- | --- |
| Species | Change Point Detected | Level of Model Success | Individual ID | 95% Credible Interval | 50% Credible Interval | 95% CI Quantile Width |
| **Deer** | Yes | Level 2 | 02.d | [ -24 , 5 ] | [ -3 , 1 ] | 29 |
|  |  | Not Successful | 10.d | [ 4 , 27 ] | [ 11 , 16 ] | 23 |
|  |  |  | 12.d | [ -4 , 35 ] | [ 10 , 25 ] | 39 |
|  |  |  | 16.d | [ -37 , 18 ] | [ -31 , -9 ] | 55 |
|  |  |  | 14.d | [ -54 , 19 ] | [ -23 , -12 ] | 73 |
|  |  |  | 15.d | [ -49 , 44 ] | [ -41 , -33 ] | 93 |
|  |  |  | 01.d | [ -29 , 68 ] | [ 60 , 65 ] | 97 |
|  |  |  | 08.d | [ -88 , 69 ] | [ 19 , 23 ] | 157 |
|  |  | Level 3 | 03.d | [ -35 , 44 ] | [ -22 , 3 ] | 79 |
|  | No | - | 04.d | - | - | - |
|  |  |  | 05.d | - | - | - |
|  |  |  | 06.d | - | - | - |
|  |  |  | 07.d | - | - | - |
|  |  |  | 09.d | - | - | - |
|  |  |  | 11.d | - | - | - |
|  |  |  | 13.d | - | - | - |
|  |  |  | 17.d | - | - | - |
| **Elk** | Yes | Not Successful | 07.e | [ -18 , -18 ] | [ -18 , -18 ] | 0 |
|  |  |  | 27.e | [ -12 , -12 ] | [ -12 , -12 ] | 0 |
|  |  |  | 06.e | [ -24 , -23 ] | [ -24 , -24 ] | 1 |
|  |  |  | 08.e | [ -25 , -24 ] | [ -25 , -25 ] | 1 |
|  |  |  | 17.e | [ -28 , -27 ] | [ -27 , -27 ] | 1 |
|  |  |  | 34.e | [ -14 , -13 ] | [ -14 , -14 ] | 1 |
|  |  |  | 35.e | [ -30 , -29 ] | [ -30 , -30 ] | 1 |
|  |  |  | 02.e | [ -34 , -32 ] | [ -33 , -32 ] | 2 |
|  |  |  | 10.e | [ -27 , -25 ] | [ -26 , -25 ] | 2 |
|  |  |  | 29.e | [ -18 , -16 ] | [ -18 , -18 ] | 2 |
|  |  |  | 33.e | [ -21 , -19 ] | [ -21 , -21 ] | 2 |
|  |  |  | 14.e | [ -14 , -11 ] | [ -13 , -12 ] | 3 |
|  |  |  | 20.e | [ -24 , -21 ] | [ -23 , -23 ] | 3 |
|  |  |  | 30.e | [ -64 , -61 ] | [ -64 , -64 ] | 3 |
|  |  |  | 32.e | [ -26 , -23 ] | [ -25 , -24 ] | 3 |
|  |  |  | 03.e | [ -11 , -7 ] | [ -10 , -9 ] | 4 |
|  |  |  | 04.e | [ -38 , -34 ] | [ -36 , -35 ] | 4 |
|  |  |  | 18.e | [ -22 , -18 ] | [ -21 , -20 ] | 4 |
|  |  |  | 23.e | [ -85 , -81 ] | [ -84 , -83 ] | 4 |
|  |  |  | 15.e | [ -24 , -19 ] | [ -22 , -21 ] | 5 |
|  |  |  | 12.e | [ -17 , -11 ] | [ -14 , -13 ] | 6 |
|  |  |  | 25.e | [ -30 , -24 ] | [ -28 , -27 ] | 6 |
|  |  |  | 28.e | [ -19 , -13 ] | [ -16 , -15 ] | 6 |
|  |  |  | 13.e | [ -21 , -14 ] | [ -18 , -16 ] | 7 |
|  |  |  | 19.e | [ -24 , -17 ] | [ -22 , -20 ] | 7 |
|  |  |  | 26.e | [ -27 , -20 ] | [ -23 , -21 ] | 7 |
|  |  |  | 11.e | [ 20 , 29 ] | [ 28 , 28 ] | 9 |
|  |  |  | 05.e | [ -34 , -24 ] | [ -31 , -29 ] | 10 |
|  |  |  | 22.e | [ -32 , -22 ] | [ -27 , -25 ] | 10 |
|  |  |  | 09.e | [ -18 , -7 ] | [ -14 , -11 ] | 11 |
|  |  |  | 21.e | [ -27 , -16 ] | [ -21 , -19 ] | 11 |
|  |  |  | 37.e | [ -23 , -12 ] | [ -18 , -15 ] | 11 |
|  |  |  | 31.e | [ -36 , -23 ] | [ -27 , -24 ] | 13 |
|  |  |  | 16.e | [ -75 , -55 ] | [ -70 , -65 ] | 20 |
|  |  |  | 36.e | [ -35 , -10 ] | [ -25 , -22 ] | 25 |
|  |  |  | 24.e | [ 13 , 50 ] | [ 34 , 46 ] | 37 |
|  |  |  | 01.e | [ -67 , 50 ] | [ -51 , -38 ] | 117 |

Table 4: Summary of MMCPM in deer and elk. If a change was detected, we categorized the ability of the model into three *a priori* levels of success based on the posterior distribution of the estimated change point centered on the known parturition event. For each individual we present the 95% and 50% credible intervals (CI) for the posterior distribution of the estimated change point centered on the true parturition event and the quantile width for the 95% CI.

|  | | | | | | |
| --- | --- | --- | --- | --- | --- | --- |
| Species | Change Point Detected | Level of Model Success | Individual ID | 95% Credible Interval | 50% Credible Interval | 95% CI Quantile Width |
| **Deer** | Yes | Not Successful | 09.d | [ -64 , -61 ] | [ -63 , -62 ] | 3 |
|  |  |  | 11.d | [ -67 , -60 ] | [ -63 , -62 ] | 7 |
|  |  |  | 10.d | [ 17 , 43 ] | [ 41 , 42 ] | 26 |
|  |  |  | 14.d | [ -43 , 4 ] | [ -19 , -13 ] | 47 |
|  |  |  | 12.d | [ -66 , 22 ] | [ -61 , -48 ] | 88 |
|  |  |  | 07.d | [ -46 , 66 ] | [ 37 , 58 ] | 112 |
|  | No | - | 01.d | - | - | - |
|  |  |  | 04.d | - | - | - |
|  |  |  | 05.d | - | - | - |
|  |  |  | 06.d | - | - | - |
|  |  |  | 08.d | - | - | - |
|  |  |  | 13.d | - | - | - |
|  |  |  | 15.d | - | - | - |
|  |  |  | 16.d | - | - | - |
|  |  |  | 17.d | - | - | - |
| **Elk** | Yes | Level 1 | 13.e | [ -6 , -4 ] | [ -5 , -5 ] | 2 |
|  |  |  | 08.e | [ -6 , -3 ] | [ -4 , -4 ] | 3 |
|  |  |  | 24.e | [ -1 , 2 ] | [ 1 , 2 ] | 3 |
|  |  |  | 14.e | [ -6 , 0 ] | [ -4 , -3 ] | 6 |
|  |  |  | 03.e | [ -6 , 1 ] | [ -1 , -1 ] | 7 |
|  |  |  | 23.e | [ -5 , 3 ] | [ -4 , -3 ] | 8 |
|  |  | Level 2 | 36.e | [ -9 , -5 ] | [ -6 , -6 ] | 4 |
|  |  |  | 26.e | [ -16 , -4 ] | [ -6 , -6 ] | 12 |
|  |  |  | 12.e | [ -7 , 10 ] | [ -6 , -6 ] | 17 |
|  |  | Level 3 | 25.e | [ -9 , 9 ] | [ 4 , 8 ] | 18 |
|  |  | Not Successful | 04.e | [ -15 , -14 ] | [ -14 , -14 ] | 1 |
|  |  |  | 22.e | [ -11 , -10 ] | [ -10 , -10 ] | 1 |
|  |  |  | 27.e | [ -15 , -14 ] | [ -14 , -14 ] | 1 |
|  |  |  | 05.e | [ -20 , -18 ] | [ -20 , -20 ] | 2 |
|  |  |  | 16.e | [ -10 , -8 ] | [ -9 , -9 ] | 2 |
|  |  |  | 28.e | [ -13 , -11 ] | [ -12 , -12 ] | 2 |
|  |  |  | 06.e | [ -14 , -11 ] | [ -12 , -12 ] | 3 |
|  |  |  | 09.e | [ 11 , 14 ] | [ 12 , 12 ] | 3 |
|  |  |  | 37.e | [ -9 , -6 ] | [ -7 , -7 ] | 3 |
|  |  |  | 11.e | [ -11 , -7 ] | [ -9 , -9 ] | 4 |
|  |  |  | 19.e | [ -65 , -61 ] | [ -62 , -61 ] | 4 |
|  |  |  | 20.e | [ -35 , -31 ] | [ -31 , -31 ] | 4 |
|  |  |  | 31.e | [ -12 , -8 ] | [ -9 , -8 ] | 4 |
|  |  |  | 34.e | [ -10 , -6 ] | [ -9 , -8 ] | 4 |
|  |  |  | 10.e | [ -40 , -35 ] | [ -36 , -36 ] | 5 |
|  |  |  | 15.e | [ -19 , -14 ] | [ -18 , -16 ] | 5 |
|  |  |  | 21.e | [ -13 , -8 ] | [ -10 , -10 ] | 5 |
|  |  |  | 35.e | [ -9 , -4 ] | [ -8 , -8 ] | 5 |
|  |  |  | 01.e | [ -57 , -50 ] | [ -51 , -50 ] | 7 |
|  |  |  | 02.e | [ -20 , -13 ] | [ -18 , -17 ] | 7 |
|  |  |  | 29.e | [ -12 , -4 ] | [ -9 , -7 ] | 8 |
|  |  |  | 32.e | [ -22 , -13 ] | [ -22 , -22 ] | 9 |
|  |  |  | 18.e | [ -18 , -8 ] | [ -11 , -8 ] | 10 |
|  |  |  | 30.e | [ -24 , -11 ] | [ -12 , -12 ] | 13 |
|  |  |  | 07.e | [ -34 , -20 ] | [ -20 , -20 ] | 14 |
|  |  |  | 17.e | [ -13 , 4 ] | [ -7 , -5 ] | 17 |
|  |  |  | 33.e | [ -18 , 1 ] | [ -17 , -17 ] | 19 |

FIGURES

**
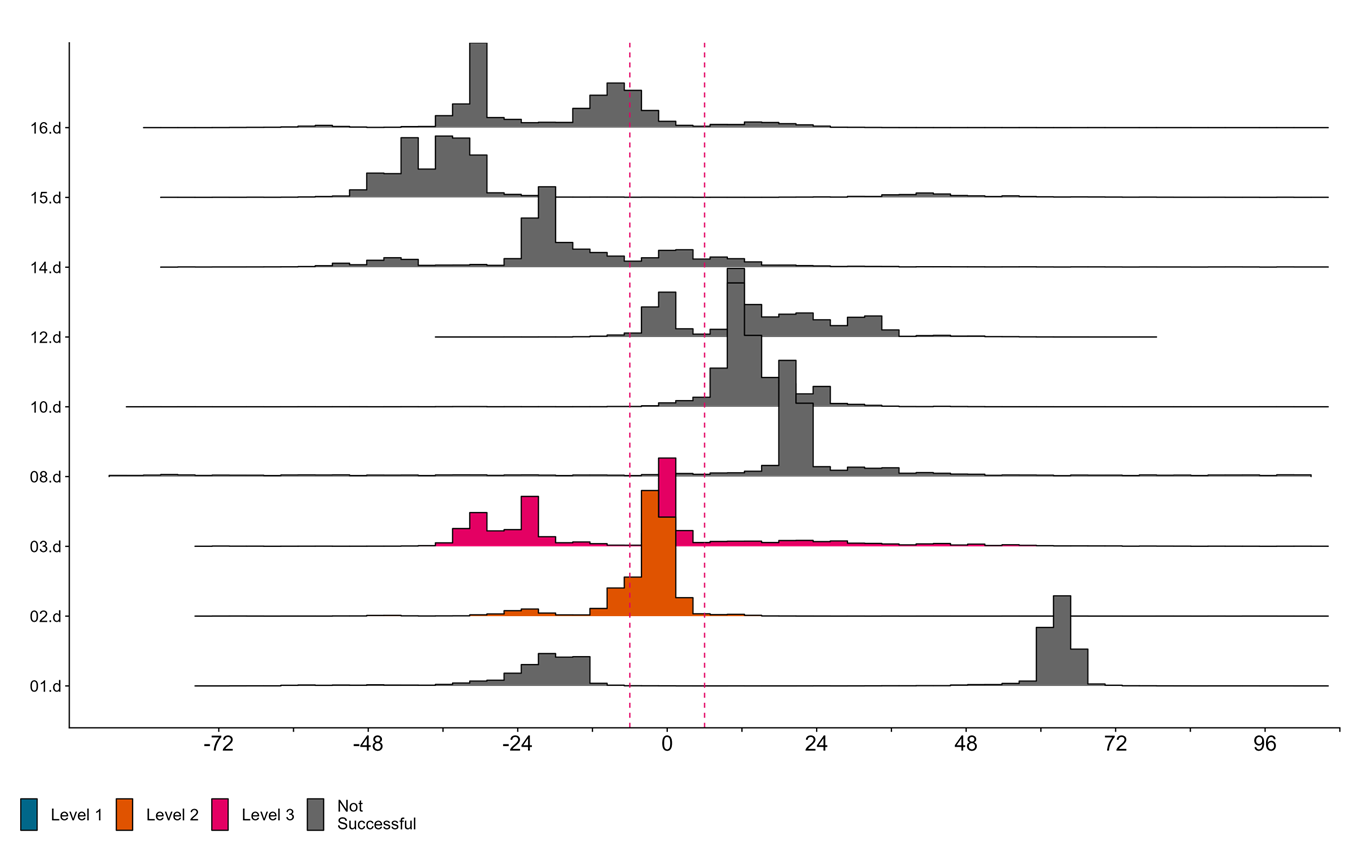
**

Figure 1: Results of the LCPM fit to deer. For each individual we present the median and the 50% and 95% credible intervals for the difference between the true parturition event and the estimated change point. The vertical, red dashed line represents ± six hours pre- and post-parturition.


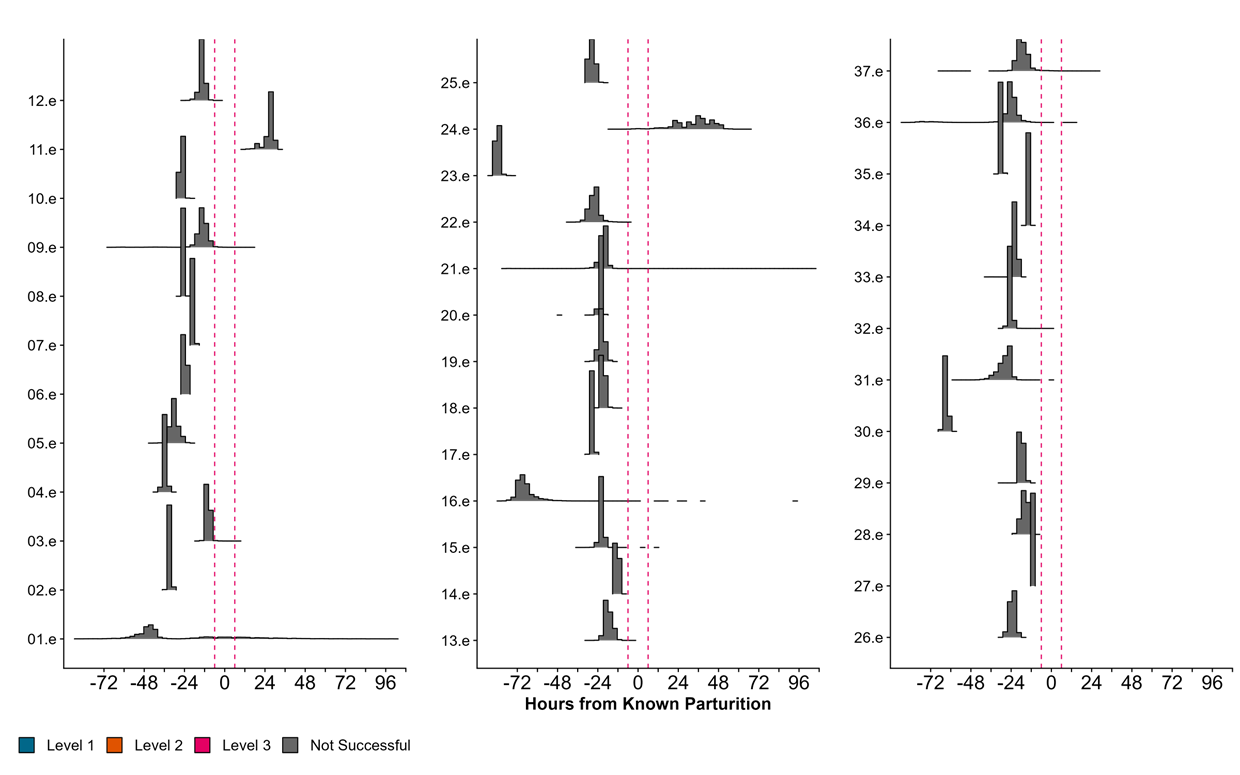


Figure 2: Results of the LCPM fit to elk. For two individuals the model failed to detect a change point and are not represented. For the 35 for which the LCPM detected a change we present the difference between the true parturition event and the estimated change point. The vertical, red dashed line represents ± six hours pre- and post-parturition.


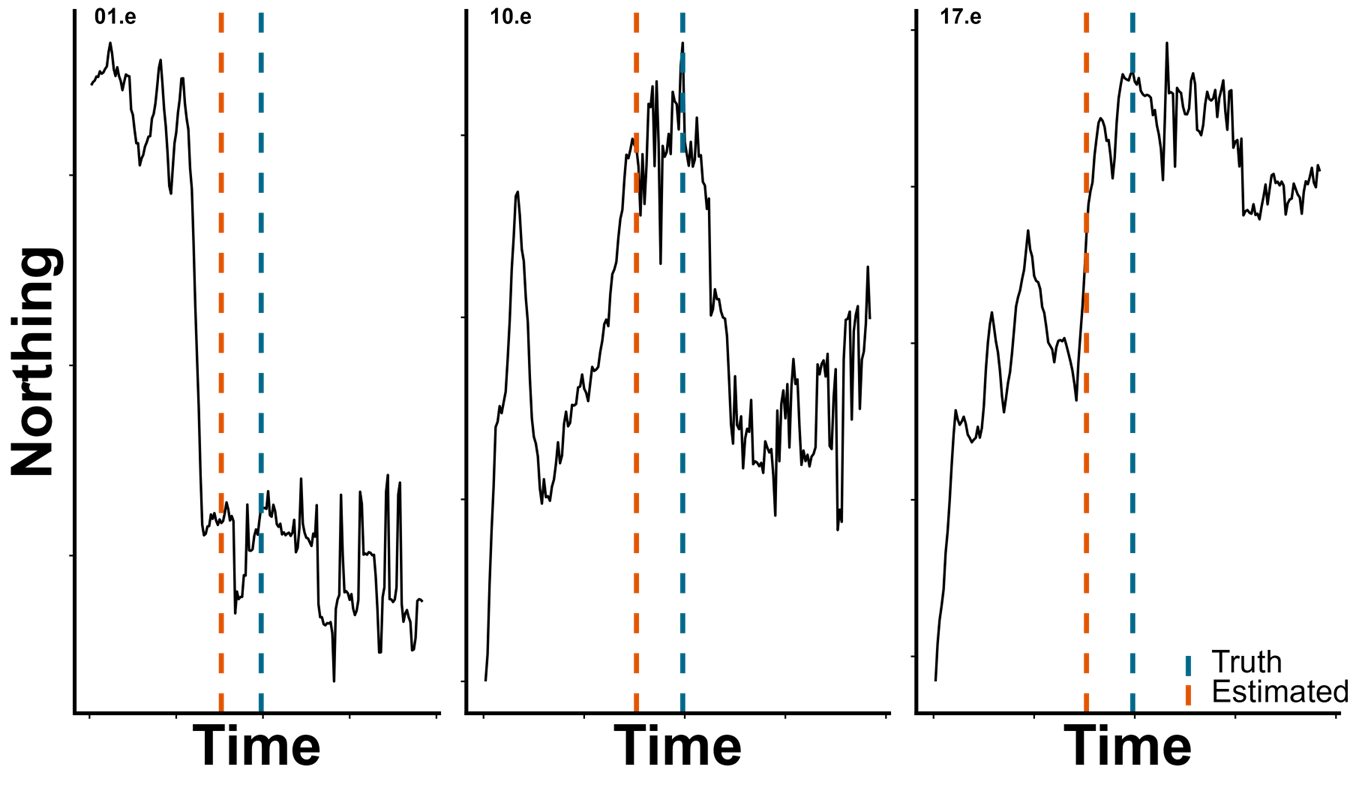


Figure 3: Examples of three elk for which the LCPM estimated a change point 12-36 hours prior to the true parturition event.


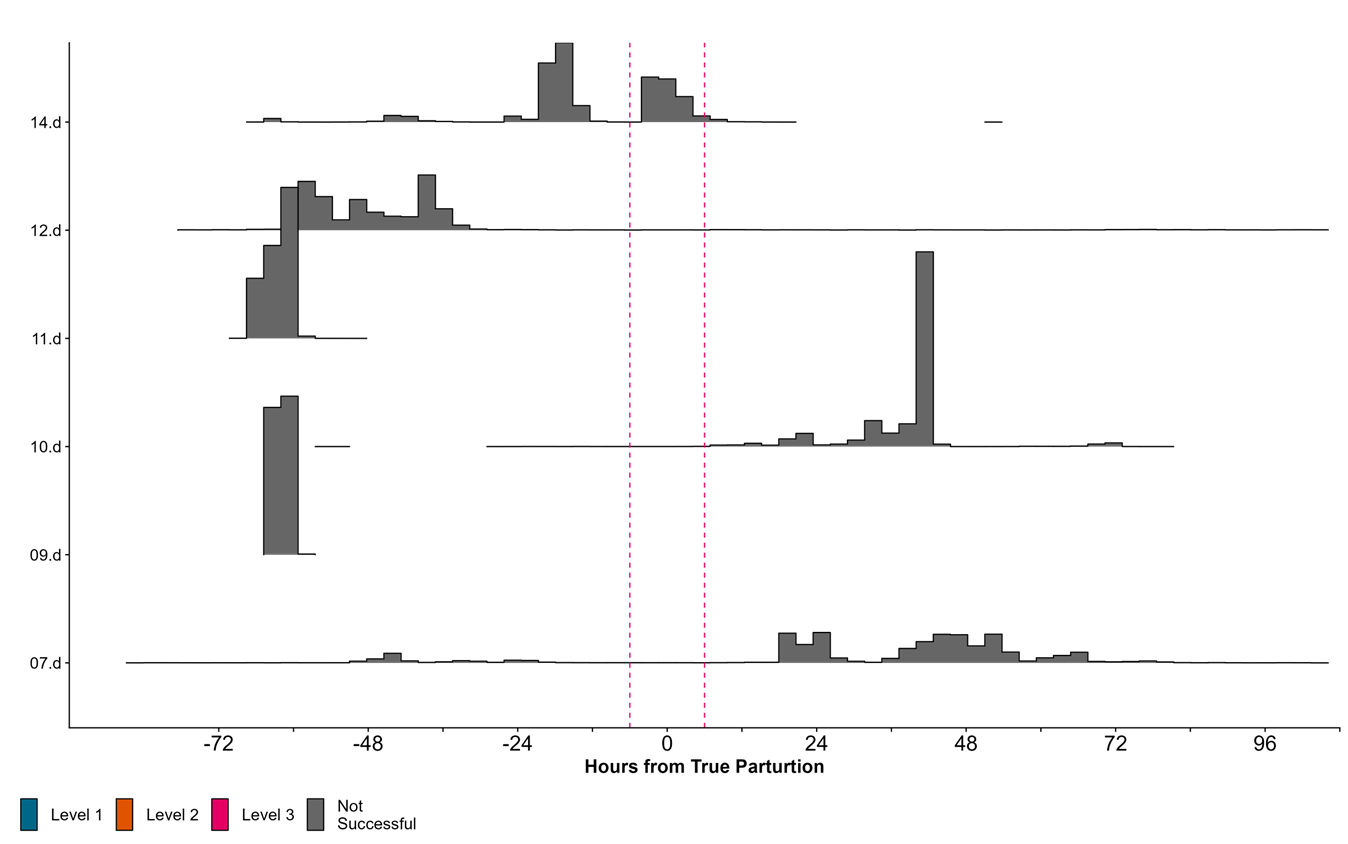


Figure 4: Results of the MMCPM fit to deer. The MMCPM failed to converge for two individuals and failed to detect a change in 9 individuals. For the individuals the MMCPM estimated a change we present the posterior distribution of the estimated change point centered on the known parturition event. The vertical, red dashed line represents ± six hours pre- and post-parturition..

*
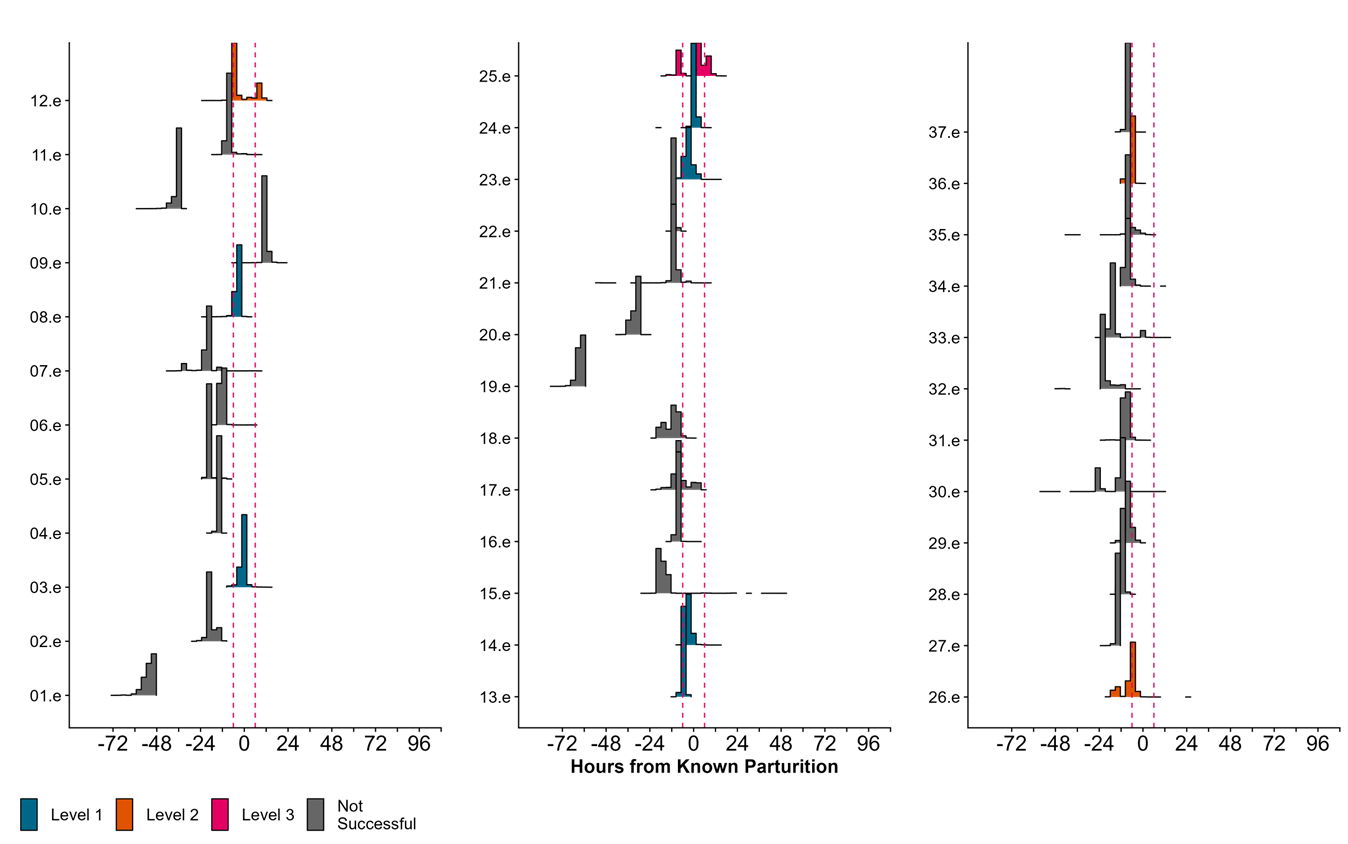
*

Figure 5: Results of the MMCPM fit to elk. We present the posterior distribution of the estimated change point centered on the known parturition event. The vertical, red dashed line represents ± six hours pre- and post-parturition. Level 1 success is denoted in blue (95% credible intervals falling between ± six hours), Level 2 success is denoted in orange (50% credible intervals falling between ± six hours), and Level 3 success is denoted in pink (median falling between ± six hours). Individuals that did not fall within the *a priori* levels of success are grey.


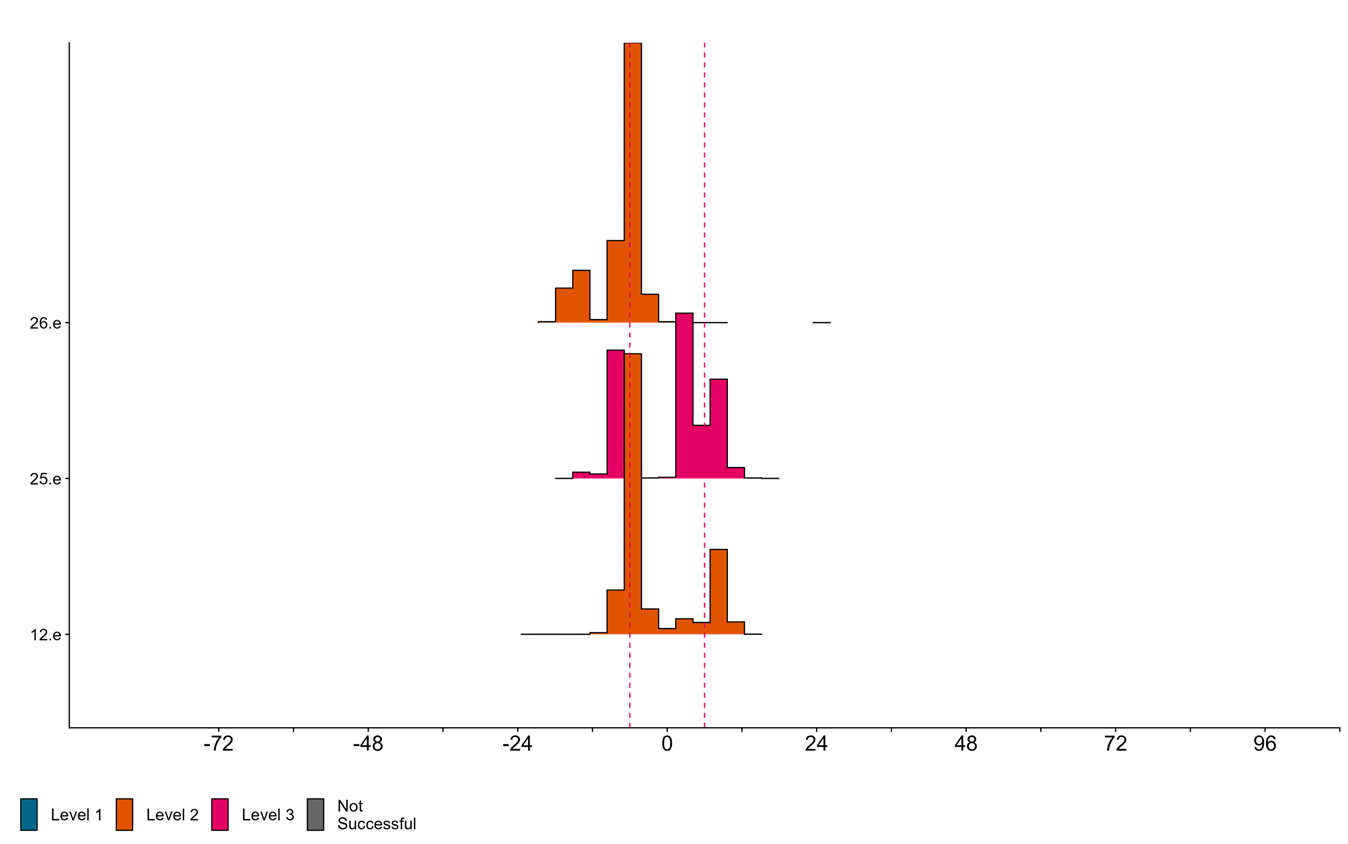


Figure 6: Examples of three elk for which the MMCPM, two of which (12.e and 25.e) exhibited bi-modal posterior distributions of the estimated change point compared to an individual where a single change was detected (26.e). The vertical, red dashed line represents ± six hours pre- and post-parturition. Level 1 success is denoted in blue (95% credible intervals falling between ± six hours), Level 2 success is denoted in orange (50% credible intervals falling between ± six hours), and Level 3 success is denoted in pink (median falling between ± six hours). Individuals that did not fall within the *a priori* levels of success are grey.
